# Automated degenerate PCR primer design for high-throughput sequencing improves efficiency of viral sequencing

**DOI:** 10.1186/1743-422X-9-261

**Published:** 2012-11-06

**Authors:** Kelvin Li, Susmita Shrivastava, Anushka Brownley, Dan Katzel, Jayati Bera, Anh Thu Nguyen, Vishal Thovarai, Rebecca Halpin, Timothy B Stockwell

**Affiliations:** 1 The J. Craig Venter Institute, 9704 Medical Center Drive, Rockville, MD, 20850, USA; 2 BioTeam Inc., 7 Derosier Drive, Middleton, MA, 01949, USA; 3 Department of Biology, University of Virginia, 485 McCormick Road, Charlottesville, VA, 22908, USA

**Keywords:** High-throughput computational degenerate PCR primer design, sequencing viral isolates

## Abstract

**Background:**

In a high-throughput environment, to PCR amplify and sequence a large set of viral isolates from populations that are potentially heterogeneous and continuously evolving, the use of degenerate PCR primers is an important strategy. Degenerate primers allow for the PCR amplification of a wider range of viral isolates with only one set of pre-mixed primers, thus increasing amplification success rates and minimizing the necessity for genome finishing activities. To successfully select a large set of degenerate PCR primers necessary to tile across an entire viral genome and maximize their success, this process is best performed computationally.

**Results:**

We have developed a fully automated degenerate PCR primer design system that plays a key role in the J. Craig Venter Institute’s (JCVI) high-throughput viral sequencing pipeline. A consensus viral genome, or a set of consensus segment sequences in the case of a segmented virus, is specified using IUPAC ambiguity codes in the consensus template sequence to represent the allelic diversity of the target population. PCR primer pairs are then selected computationally to produce a minimal amplicon set capable of tiling across the full length of the specified target region. As part of the tiling process, primer pairs are computationally screened to meet the criteria for successful PCR with one of two described amplification protocols. The actual sequencing success rates for designed primers for measles virus, mumps virus, human parainfluenza virus 1 and 3, human respiratory syncytial virus A and B and human metapneumovirus are described, where >90% of designed primer pairs were able to consistently successfully amplify >75% of the isolates.

**Conclusions:**

Augmenting our previously developed and published JCVI Primer Design Pipeline, we achieved similarly high sequencing success rates with only minor software modifications. The recommended methodology for the construction of the consensus sequence that encapsulates the allelic variation of the targeted population and is a key step prior to designing degenerate primers is also formally described.

## Background

Organisms that evolve rapidly have the selective advantage of quickly adapting to their host, or surroundings. For example, a few amino acid changes on the surface protein (hemagglutinin) of the influenza virus are sufficient to evade the host’s antibody detection within the time span of a single season [1]. To effectively genotype and monitor the genome of a rapidly evolving and heterogeneous population, it is important to have the capability to amplify the nucleic acid sequences from samples taken from that population as the genotype is shifting. As the viral sequencing projects have increased at JCVI over the past few years, a high-throughput mechanism to rapidly select PCR primers with a high probability of success was necessary. In this paper, we describe the modifications made to our high-throughput primer design pipeline, previously developed for human re-sequencing [2]. We describe the results for 8 out of the 15 viruses that have had PCR primers designed for: measles virus, mumps virus, rubella virus, human parainfluenza virus 1 and 3, human respiratory syncytial virus A and B and human metapneumovirus. PCR primers for the remaining viruses were also successfully designed using the same degenerate primer design pipeline, but were not included in the results and discussion, because a few primers from the entire computationally designed set were modified to suit project specific laboratory PCR requirements. Thus, the utilized primer pairs were not completely true to the algorithm’s recommendations. The excluded viruses were human coronavirus, feline coronavirus, canine coronavirus, SARS coronavirus, human adenovirus, human Norwalk virus, and influenza viruses (human influenza A and B, and avian influenza A).

The use of degenerate primers to selectively amplify a set of closely related sequences is periodically found in literature. Previous work on computational tools have included, CODEHOP [3] which designs degenerate primers based on conserved amino acid sequences and HYDEN [4] which tries to maximize the coverage of a degenerate primer pair with a minimal amount of degeneracy. For viral sequences, an amino acid based methodology of CODEHOP would introduce a larger set of degenerate bases than would be necessary to successfully amplify a set of isolates, thus unnecessarily increasing the potential for non-specific priming and alternative products. HYDEN’s objective focuses on a primer pair for a family of genes, rather than the whole genome sequencing of a potentially heterogeneous sample of same species viruses in a high-throughput environment.

The goal of JCVI’s degenerate primer design pipeline is to maximize the success rate of selected primer pairs, given a set of sequences that are assumed to represent the target population. It is not only important to decide which bases to make degenerate, but also how many and which combinations will lead to successful PCR. The high throughput features of the JCVI’s degenerate primer design pipeline that have been inherited from the original non-degenerate version, include dynamic tiling, an overlapping non-fixed amplicon set across the entire target genome, as well as the capability to predict primer pairs that have very high success rates under one of either of two amplification protocols: “standard” or “high GC”. Dynamic tiling allows the selection of an amplicon set to be dependent on the landscape of features on the template. By parameterizing the minimum, maximum, and optimal amplicon overlap and amplicon sizes, the JCVI primer design pipeline crawls across a template based on the last amplicon selected, thus minimizing the number of successful amplicons necessary to cover the genome while only selecting amplicons predicted to be successful. This exhaustive search is not possible with fixed tiling.

The necessity of designing highly successful primers for a high-throughput directed sequencing environment is a consequence of the prohibitively high labor costs required to manually reprocess failures by tuning PCR conditions. The work we present here utilizes two laboratory protocols based on our previous publication to achieve a comparable level of success.

In this paper, the results of the degenerate primer design, the augmentation of the previously published, high-throughput PCR primer design software [2], and the methodology used to construct the reference degenerate consensus sequence are described.

## Results and discussion

### Targeted viruses

The degenerate primer design pipeline was used to design primers for multiple viral projects across 15 different DNA and RNA viruses including both segmented and non-segmented viruses. The results for 8 different non-segmented viruses are presented here. The size of these genomes varied from 9kb to 15.6kb with various degrees of sampled genomic sequence variation and GC content. The summary statistics for the primers designed for the 8 different non-segmented viral genomes using the two different PCR protocols described as the “standard” and “high GC” protocols are presented in Table 1. The standard and high GC protocols are described briefly in the Materials and Methods section.

**Table 1:** Summary statistics for targeted viruses and sequencing results

**Virus type**	**Forward success rate (%)**	**Reverse success rate (%)**	**Average success rate (%)**	**Number of reactions successful**	**Number of reactions total**	**Ambiguity in consensus (%)**	**GC content of consensus sequence (%)**	**Number of sequences in consensus**	**Number of amplicons designed**	**Minimum coverage**	**BioProject ID**
**HPIV-1**	98.95	100.00	99.47	378	380	5.10	34.64	5	95	3x	PRJNA73053
**HPIV-3**	98.42	97.89	98.16	373	380	7.77	31.24	5	95	3x	PRJNA73055
**MeV**	93.41	95.05	94.23	1738	1860	10.76	41.94	25	91	3x	PRJNA69913
**MuV**	73.85	73.68	73.77	1963	2661	9.28	37.97	32	96	3x	PRJNA73019
**RUBV-G1**	80.05	78.63	79.34	3556	4882	6.07	66.75	11	96	2x	PRJNA70479
**RUBV-G2**	71.52	69.24	70.38	1478	2100	13.13	61.37	5	96	2x	PRJNA70479
**HRSV-A**	91.32	89.96	90.64	3004	3332	6.81	30.25	3	50	2x	PRJNA73049
**HRSV-B**	91.37	90.86	91.11	2436	2682	4.12	31.79	3	59	2x	PRJNA73049
**HMPV-A**	90.99	88.82	89.91	1737	1932	9.25	31.53	6	42	1x	PRJNA73051
**HMPV-B**	86.16	87.04	86.60	788	910	7.95	32.68	5	36	1x	PRJNA73051

Table 1 contains the actual sequencing success rates for each targeted viral consensus sequence and the relevant information regarding each consensus sequence’s construction. The median amplicon coverage for all viruses is approximately 3x, however in many cases the ends of the genomes had a lower minimum coverage, i.e.,1x or 2x, due to the inability to select primer beyond the end of the available sequence. Dynamic tiling produced an even tiling of amplicon coverage across all genomes.

Primers designed using the standard protocol targeted organisms with less than 50% GC content. These included the human parainfluenza virus (HPIV-1 and HPIV-3), measles virus (MeV), mumps virus (MuV), human respiratory syncytial virus (HRSV-A and HRSV-B) and human metapneumovirus (HMPV-A and HMPV-B). For organisms with GC content exceeding 50%, e.g., rubella virus (RUBV-1 and RUBV-2), the high GC protocol was used.

### Consensus sequence construction

Amplicons were designed to cover the entire genome for all viruses, so only full length complete sequences available from NCBI’s Viral Genomes [5] were used to generate the consensus sequences. The number of sequences used to generate the consensus sequence and the percent of degenerate bases across the constructed consensus sequences are described in Table 1. The number of sequences collected to construct each consensus sequence ranged from 3 (for HRSV-A and HRSV-B) to 32 (for MuV).

For each viral type, a single consensus file was generated with a target of less than 10% degenerate bases. With two exceptions, the resultant percent ambiguity across the constructed consensus sequence ranged from 4.12% for HRSV-B to 9.28% for MuV. If a single consensus could not be generated with less than 10% ambiguity, multiple consensus sequences were constructed based on sequence similarity-based clustering results. The two exceptions were measles virus (MeV) and rubella virus (RUBV-G2) which had percent ambiguities exceeding 10%.

For measles virus, the allele frequencies for multi-allelic positions were not filtered to remove the less dominant allele frequencies, even when the percentage of degenerate bases across the constructed consensus sequence exceeded 10%. Even though the less dominant allele frequencies were below the expected threshold for sequencing error, because the percent degeneracy across the consensus sequence was very close to 10%, it was decided that stratifying the sequences and generating multiple consensus sequences may not be cost effective. The total success rate was not expected to be significantly impacted.

**Figure 1:**
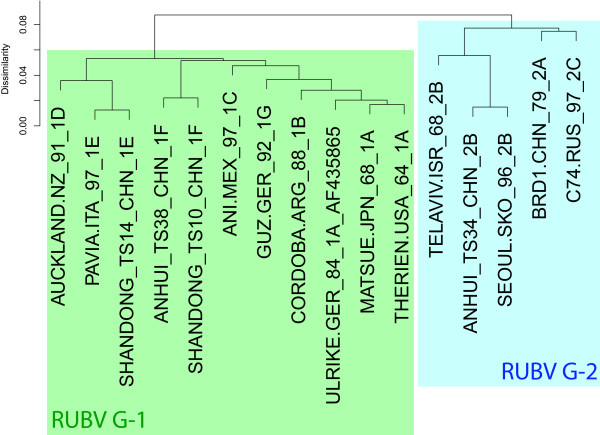
**Dendrogram representing the relationship between sequenced Rubella Virus (RUBV) genomes.** The Rubella sequences were divided into two groups, RUBV-G1 (green) and RUBV-G2 (blue), to reduce the percent ambiguity across their consensus sequences. The hierarchical clustering methodology used was complete linkage.

For the rubella virus, the input sequences were stratified into two genotypes because the total percentage of degenerate bases in the initially constructed consensus sequence was 21% (see Figure 1). After computational stratification, two sets of sequences were used as input to generate consensus sequences for genotype 1 (G1) and genotype 2 (G2), based on 11 and 5 sequences, respectively. This reduced the percent degeneracy across the consensus sequence to 6.07% and 13.13%, for G1 and G2, respectively. G2 contained many multi-allelic positions that were not filtered. At an allelic frequency of 20% (1 out of 5), it was not clear whether these should be attributed to sequencing error within the input sequences, so the variations were retained just to be conservative. Degenerate primers were computed independently for the G1 and G2 consensus sequences, and then redundant primer pairs between the two computes were removed.

**Figure 2:**
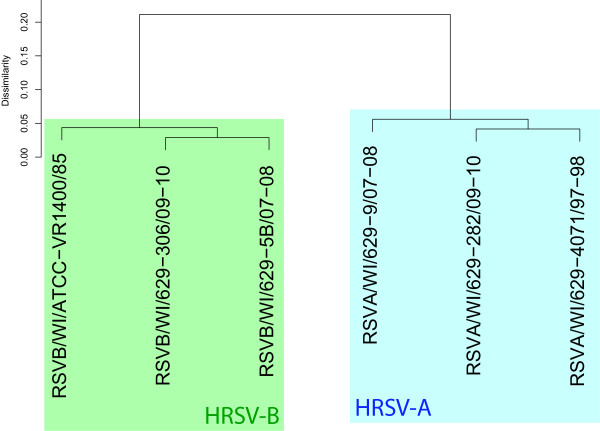
**Dendrogram based on sequence similarity for Human respiratory syncytial virus (HRSV) genome.** The 6 HRSV genomes were divided based on sequence similarity into two clades, HRSV-A (blue) and HRSV-B (green). Two consensus sequences were constructed. The percent dissimilarity between the two clades was approximately 20%.

For human respiratory syncytial virus (HRSV), separate sets of primers were designed for HRSV-A and HRSV-B since there was more than 20% sequence dissimilarity across the entire genome with 24% ambiguity (see Figure 2). Splitting the sequences into two subsets ensured that the sequences in each subset were less than 10% dissimilar. The sequences in the subset HRSV-A differed by 5.5% and those in HRSV-B differed by 4.5%, with a percent ambiguity of 6.8% for HRSV-A and 4.1% for HRSV-B.

For human metapneumovirus (HMPV), there appeared to be two distinct clades, between which they were approximately 20% dissimilar. These initial sequences were split into two clades of HMPV-A, with 9.25% ambiguity and HMPV-B with 7.95% ambiguity. Separate primers pairs were then designed for the two clades independently.

### Coverage depth

For all viral genomes, amplicons were designed with an intended coverage depth of 2x or 3x; with the exception of HMPV-A and HMPV-B, which had a coverage depth of only 1x. Multiple depths of coverage increase the likelihood of successfully amplifying the targeted region at least once because when a second pair of candidate primers is selected, the primer design pipeline ensures that the second primer pair will not overlap any of the previously selected primer pairs. In a high-throughput sequencing environment, all wells in a plate are processed, so additional amplicon coverage was generated if any empty wells remained. If the number of amplicons designed exceeded the number of wells on a plate, amplicons were computationally reduced to a smaller subset so that every targeted region was covered by at least one amplicon. Projects also differed by their sequencing requirements, thus resulting in varying amplicon numbers and coverage depth, depending on each virus type.

### Calculating success rates

Determining the success of degenerate primer design is a more complicated process than that of standard non-degenerate primer design. Not only does the degenerate primer design algorithm need to accurately model and predict the outcome of PCR with a heterogeneous population of primer pairs, but the constructed degenerate consensus sequence needs to sufficiently represent the targeted genome’s population. The latter can be detrimentally impacted by a lack of available sequence information, or the potential for heavily biased sequencing favouring strains specifically studied by a single, or few laboratories.

**Figure 3:**
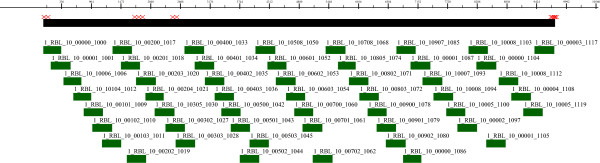
**Dynamically tiled layout of the amplicons across the targeted genome.** This is an example of the tiling output from a single primer design run performed on RUBV-G1. Each green rectangle represents an amplicon. The long black bar represents the targeted region and the red X’s, above the targeted region, represent locations where 2x coverage could not be achieved. Amplicon lengths for RUBV-G1 and RUBV-G2 using the high GC protocol were targeted to be between 300–350 bp.

The success rates provided in Table 1 represent the average success rates for each virus type as a percentage of all sequencing reactions performed. However, since the effect of isolate is confounded inside of the average success rate, to gain a deeper understanding of the success rate of primer pairs across isolates, a graded approach was taken. This was necessary because if a primer pair was successful at a low percentage across all samples, then it was possible that the primers did not match the genotype of the isolate, rather than a poorly selected primer pair based on the primer design algorithm alone. For each primer pair and isolate, sequencing was performed in both the forward and reverse directions. If a sequence was recovered for an isolate and primer pair (under standard expectations of high quality values, length, etc.) then that sequencing direction was considered a success. Success rates were tallied for forward, reverse, and then averaged between both sequencing directions. These per primer pair success rates were then graded at cutoffs of greater than 25, 50, 75, 85 and 90 percent of isolates. (See Table 2) This cumulative and graded approach was necessary to distinguish between amplification failures that were either isolate or target region specific. Note that these are actual, not predicted, success rates calculated based on laboratory experiments, not predicted success rates based on *in silico* computations. Furthermore, because of the fairly uniform amplicon coverage across all genomes, e.g., Figure 3, the reported success rates should not be inflated by any redundant stacking of amplicons over easy-to-amplify loci. Table 2 contains the forward, reverse, and average success rates for each viral consensus sequence. For each directional set of statistics, the sequencing success rate was computed across all isolates. For example, for HRSV-A, 94% of all primers designed had greater than 75% of the isolates successfully sequenced.

**Table 2:** Designed primer pair success statistics by cumulative isolate success rate

	**Forward average success rate**					**Reverse average success rate**					**Total average success rate**				
**Virus Type**	**>25%**	**>50%**	**>75%**	**>85%**	**>90%**	**>25%**	**>50%**	**>75%**	**>85%**	**>90%**	**>25%**	**>50%**	**>75%**	**>85%**	**>90%**
**HPIV-1**	0.99	0.99	0.99	0.99	0.99	1.00	1.00	1.00	1.00	1.00	1.00	1.00	0.99	0.99	0.99
**HPIV-3**	0.99	0.99	0.98	0.98	0.98	0.99	0.99	0.97	0.97	0.97	0.99	0.99	0.98	0.97	0.97
**MeV**	0.99	0.97	0.95	0.88	0.88	1.00	0.99	0.97	0.90	0.90	1.00	1.00	0.95	0.89	0.87
**MuV**	1.00	0.98	0.90	0.88	0.60	1.00	0.97	0.89	0.85	0.54	1.00	0.97	0.89	0.85	0.63
**HRSV-A**	1.00	0.98	0.94	0.86	0.74	0.98	0.98	0.94	0.86	0.74	1.00	0.98	0.94	0.90	0.76
**HRSV-B**	1.00	1.00	0.97	0.83	0.76	1.00	1.00	0.97	0.84	0.79	1.00	1.00	0.98	0.91	0.76
**HMPV-A**	1.00	0.95	0.95	0.93	0.83	0.98	0.93	0.93	0.90	0.81	1.00	0.95	0.93	0.88	0.81
**HMPV-B**	0.94	0.94	0.94	0.83	0.83	0.97	0.94	0.94	0.83	0.83	0.94	0.94	0.94	0.83	0.80
**AVERAGE**	**0.99**	**0.98**	**0.95**	**0.90**	**0.83**	**0.99**	**0.97**	**0.95**	**0.90**	**0.82**	**0.99**	**0.98**	**0.95**	**0.90**	**0.82**
**MEDIAN**	**0.99**	**0.98**	**0.95**	**0.88**	**0.83**	**0.99**	**0.98**	**0.95**	**0.88**	**0.82**	**1.00**	**0.98**	**0.94**	**0.90**	**0.80**
RUBV-G1 *	0.94	0.85	0.73	0.49	0.27	0.90	0.84	0.71	0.51	0.17	0.94	0.85	0.70	0.46	0.26
RUBV-G2 *	0.95	0.90	0.82	0.55	0.55	0.93	0.86	0.75	0.52	0.52	0.95	0.90	0.75	0.55	0.55
**AVERAGE (high GC)**	**0.94**	**0.88**	**0.77**	**0.52**	**0.41**	**0.92**	**0.85**	**0.73**	**0.52**	**0.35**	**0.94**	**0.88**	**0.73**	**0.51**	**0.40**
**MEDIAN (high GC)**	**0.94**	**0.88**	**0.77**	**0.52**	**0.41**	**0.92**	**0.85**	**0.73**	**0.52**	**0.35**	**0.94**	**0.88**	**0.73**	**0.51**	**0.40**

* These viruses have a GC content exceeding 67%.

To separate PCR failure bias due to the GC content of the genome alone, the overall success rates of primer pairs were calculated for high GC and standard protocols, separately. For the standard protocol, the overall forward, reverse and averaged sequencing success rates of primer pairs were 83%, 82% and 82% respectively, for over 95% of the isolates. However at greater than 75% of the isolates, there was a consistent 95% primer pair success rate. For mumps virus, most of the primer failures were recorded in the V/P protein region which has RNA editing (with the insertion of non-template G) and in the variable region of the SH gene. A consistent pattern in the plate location of the primer failures was also discovered, which could be an issue related to laboratory conditions and not due to the design of the primers.

For the high GC protocol, the three sequencing success rates of primer pairs were lower than expected. For greater than 75% of isolates, success rates were 77%, 73% and 70% for the overall forward, reverse and total sequencing success rate, respectively. There did not appear to be a relationship between the percentages of ambiguity in the consensus sequence and the success rates of the designed primers (simple linear regression, adjusted R^2^ = 0.167). This lack of correlation could be a result of the few sequences represented by the degenerate consensus sequence poorly representing the isolates very accurately.

Designed primer pair sequences and success rates are available as Additional file 1.

## Conclusions

The high-throughput degenerate PCR primer design pipeline has been very successful in providing the basis for high-quality viral sequencing results and for minimizing costs associated with labor, reprocessing, and genome finishing. The modular architecture of the primer design software has made it possible to readily integrate new features into the pipeline. As a result, the augmented primer design software, when coupled with the RT-PCR modified, production-verified, prior PCR laboratory protocols, serves as a powerful tool for high-throughput degenerate primer design, thus enabling the quick and effective sequencing of diverse viral strains. The importance of proper degenerate consensus sequence construction, a key input into the process, is described and the success rate of the designed primers supports the effectiveness of these combined methodologies.

## Materials and methods

### Modifications to JCVI’s primer design pipeline

#### Inputs

The input into the degenerate primer design pipeline is a consensus reference sequence of the targeted genome. This consensus sequence is used as a reference for detecting alternative products and as a template from which primer pair candidates are selected. For segmented viruses, or if only a subset of the entire genome is targeted, a subset may be specified as the template, but the entire genome sequence is still required as a reference for detecting potential alternative amplification products.

The generation of the consensus sequence with ambiguity codes may be established by first generating a multiple sequence alignment (MSA) with a tool such as Clustalw [6] or Muscle [7] and then using a MSA-to-consensus generator such as cons from EMBOSS [8], or one of the applications from ANDES [9]. If the sequence diversity of the targeted population or genomic region is significant, then it is recommended to use a tool, such as ANDES, to determine if it is necessary to stratify the population and also filter positions of potential variance by various means. This is described later in the Material and Methods subsection, “Constructing the consensus sequence with ANDES”.

#### Outputs

The output is a set of degenerate primer pairs and their theoretical amplicons based on the consensus reference sequence. A variety of supplemental design information is also generated. Among them is a summary file of critique results including information such as primer and amplicon location on the reference, their melting temperatures and primer dimer calculations along with the sequences of the primers. An Adobe Portable Document Format (PDF) file, which contains a visualization of the tiling layout of the amplicons across the targeted genome, is also generated along with the summary file (Figure 3).

#### Algorithm

The core architecture for primer design has remained unmodified from its original form. The Coverage Manager (CM) component determines a dynamic tiling path across the genome based on the results of the Primer Critiquor (PC) component’s examination of candidate primer pairs. The PC component consists of a series of tests, or critiques, that determine whether a primer pair will be successful based on empirical evidence from previous sequencing experiments. The CM component utilizes primer3 [10] to generate a list of candidate primer pairs, however because primer3 will return an error message if ambiguity codes are found on the user-specified template, an extra step is performed to convert ambiguity codes into N’s. After primer3 has generated a list of primer pair candidates with embedded N’s, the CM remaps the N’s back to their original ambiguity code and passes the degenerate primer pair candidates to the PC component for additional critiquing.

The following critique modules, utilized by the PC component, were modified to assess the anticipated success of primer pair candidates with degenerate base positions.

#### Alternative amplification detection

Instead of using BLAST [11], with a small word size, to search for primer binding sites on the reference consensus genome, dreg, from EMBOSS, is utilized. dreg uses regular expressions, a highly flexible pattern recognition specification, for sequence matching. Based on the conditions of the laboratory protocol utilized in the high-throughput sequencing laboratory, it was determined that the binding of the 11 base pairs of the 3’ end were sufficient for the polymerase to initiate synthesis of the copy strand. For this reason, only the 11 base pairs of the three prime end of the degenerate primer sequence are specified in the regular expression pattern. Detected primer binding sites are then analyzed to determine whether an alternative product will be formed, based on detected primer binding site orientation and pair-wise distances.

#### Primer dimer detection and melting temperature calculations

For each degenerate sequence in a primer pair candidate, a list of disambiguated sequences is generated by enumerating all possible non-degenerate primer sequences that can be represented by each degenerate sequence. Self and pair-wise primer dimer predictions are calculated within and between the disambiguated primer sequence lists, respectively. Primer melting temperatures are computed by averaging the nearest neighbor thermodynamic calculations [12] of the melting temperatures of the disambiguated sequences derived from the degenerate sequence.

### Constructing the consensus sequence with ANDES

The construction of the template and/or reference consensus sequence with ambiguous bases is a critical step prior to executing the degenerate primer design pipeline. Unlike using standard, i.e. non-degenerate, primers where the analyst must assume that the primers are binding to conserved regions surrounding the target, the necessity of degenerate primers presumes that the target population is sufficiently heterogeneous such that there are insufficient conserved regions to allow standard primers to successfully bind and amplify all samples from the population. Degenerate primer design allows for a mixed population of primer pairs to be simultaneously available during the PCR reaction, thus increasing the likelihood of successfully targeting an unknown sample from a heterogeneous population. A properly designed consensus sequence, or set of consensus sequences, will represent this heterogeneous population, maximizing the likelihood of attaining PCR products for all samples, while at the same time, minimizing the potential for alternative products. The number of alternative products may exhibit a quadratic relationship with the number of unique primer sequences involved in the PCR. Thus, it is a goal to minimize the number of degenerate bases in each primer sequence, while maximizing the proportion of the target population the primers specifically bind to. The end effect is to limit the number of degenerate bases per primer to no more than 4, but preferably less than 3 bases.

ANDES tools provide the analyst with two key methodologies to support minimizing the number of degenerate bases utilized, while maximizing the proportion of the population targeted: abundance filtering and sample stratification. Ultimately, the targeted percent of degenerate bases in the consensus sequence should be no more than 10%, but ideally less than 8%.

Abundance filtering in ANDES can be performed using several techniques to reduce the number of degenerate bases introduced into the consensus sequence. When filtering is applied, the analyst assumes that not all point mutations represented in the set of sequences acquired from the population are equally important. Some polymorphisms may be due to sequencing error, while others may only represent an insignificant subpopulation. Given a MSA constructed from a set of sequences determined to be a reasonable representation of the population, ANDES computes the distribution of nucleotides for each position. A nominal filter or percentage filter can be applied to remove the presence of alleles that are not represented at or above a user-specified frequency or percentage threshold, respectively. For example, the degenerate base W is required if both the alleles A and T exist in the population for a specific position. However if 2% of the samples are A’s, and 98% are T’s, applying a 4% percentage filter would change the W to the non-degenerate base, T, if the analyst believes that this polymorphism was actually due to sequencing error. Alternatively, a statistical filter, combining both frequency and percentage, may be applied. This utilizes the binomial distribution to determine whether an allele would be detected upon resampling to the same depth based on a user-specified confidence, i.e. alpha. Lastly, an optimization-based filter is also available, in which the analyst specifies the maximum percentage of degenerate bases to be allowed in the consensus sequences, and the algorithm iteratively removes alleles starting with the ones with the lowest proportion across the samples, until the maximum percentage of degenerate bases have been achieved. If these filtering techniques either remove alleles that need to be targeted, or do not reduce the number of degenerate bases in the constructed consensus sequence sufficiently, then sample stratification will be necessary.

The goal of sample stratification is to reduce the total variation in a combined population by placing individuals into clusters which contain less intra-cluster variance than the entire population. The more a difference between clusters can be used to separate the clusters, the less variation will exist within each of the formed clusters with respect to the entire population. This stratification, i.e. clustering, can be performed with ANDES. Given a MSA, ANDES can be used to compute a distance matrix and dendrogram, based on sequence similarity, which can then be used to generate clusters given a user-specified cutoff. The maximum intra-cluster percent dissimilarity cutoff that works well is approximately 10%. When the percent similarity within a cluster (or undivided set of sequences) falls below 90%, the number of degenerate bases that must be introduced into the consensus sequence becomes too high. For each cluster that is created to reduce the amount of intra-cluster dissimilarity, a separate consensus sequence is subsequently generated to represent that subpopulation.

When determining an appropriate threshold for any of these methodologies, it is important to keep in mind the number of sequences included, since the confidence of real polymorphisms existing in the population can only be attained with sufficient sample size. A general landscape of variation can be ascertained with a plot of normalized entropy versus location. With sufficient conserved regions flanking highly variable regions, and a sufficiently large amplicon size, regions with localized high rates of variation do not generally introduce difficulty in primer pair selection due to the primer designer’s dynamic tiling capabilities.

### Computational degenerate primer design parameters

The JCVI primer design pipeline automatically detects sub-regions of the targeted genome sequence that may use the standard protocol, or must use the high GC protocol. Once the sub-regions have been separated, the proper primer design parameters are utilized for the design and selection of primer pairs.

Key parameters shared between the standard and high GC protocols include: optimal/minimum amplicon dynamic tiling overlap of 100/100 bp, and optimal/minimum/maximum primer lengths of 20/18/25 bp, low complexity filter of 80%, primer binding site hairpin detection at >14 stem hydrogen bonds and <11 bp loop circumference size, maximum internal primer dimer of 8 bp and maximum end primer dimer of 3 bp.

Key parameters where the two protocols differed include: minimum/maximum amplicon melting temperatures of 68°/81.5°C (standard) and 85°/95°C (high GC), optimal/minimum/maximum amplicon sizes of 650/600/700 bp (standard) and 325/300/350 bp (high GC), and maximum amplicon dynamic tiling overlap of 550 bp (standard) and 300 bp (high GC). (MeV was the only exception. Its primer design utilized a project specific optimal/minimum/maximum amplicon size of 750/550/800 bp to reduce the number of amplicons necessary.)

Amplicon sizes were selected to be shorter than sequencing read length to achieve full bi-directional coverage, increasing the accuracy of downstream computational variation detection and decreasing the necessity for manual review for indeterminate base calls.

### RT-PCR

For both the standard and the high GC protocols, reverse transcription PCR (RT-PCR) was performed using Qiagen One Step RT-PCR Kit (cat# 210212). Each viral sample underwent RT-PCR reactions with 96 pairs of designed primers in a 96-well format. Qiagen One Step RT-PCR was set up in 10μl reactions containing 0.6μl water, 1.6μl buffer (Qiagen), 1.6μl Q solution (Qiagen), 0.3μl dNTP (Qiagen), 0.3μl enzyme (Qiagen), 0.4μl RNaseOut (Invitrogen), 2.2μl undiluted RNA, and 3μl primers (1.6μM, forward and reverse mixed). An additional 1.6μl of Q solution was added to each RUBV-G1 and RUBV-G2 reactions due to the high GC content of the genome, while for the other genomes, an additional 1.6μl of water was added to each reaction. Amplification was done in 96-well format on an MJ Research DNA Engine Tetrad 2 thermal cycler with the following cycling conditions: 1 cycle of 50°C (30 min); 1 cycle of 95°C (5 min); 35 cycles of 94°C (30 sec), 55°C (30 sec), and 72°C (1 min); 1 cycle of 72°C (10 min); hold at 4°C.

After amplification, 2μl of each reaction was run on a 1% agarose gel for 1 hour to visualize bands and confirm amplification. Unincorporated dNTPs were dephosphorylated and excess primers were removed from products using a SAP/Exo I reaction containing 0.5 units of shrimp alkaline phosphatase (USB Corporation), 1.0 unit of exonuclease I (USB Corporation) and molecular biology grade water. The cycling program was: 1 cycle of 37°C (60 min) and 1 cycle of 72°C (15 min).

### Sequencing

Sanger sequencing reactions were performed on each RT-PCR product on a standard high-throughput sequencing system using Big Dye Terminator v3.1 (Applied Biosystems) with M13 sequencing primers (Forward primer: TGTAAAACGACGGCCAGT; Reverse primer: CAGGAAACAGCTATGACC). Dye terminators were removed by ethanol precipitation and sequences were obtained with a 3730xl DNA Analyzer (Applied Biosystems).

Raw sequence data was computationally trimmed to remove any primer-derived sequence, as well as low quality sequence. Genome sequences were assembled using JCVI’s internally developed assembly software named “FLAP” (unpublished) that is based on *minimus,* which is a component of the open-source AMOS project [13] (http://amos.sourceforge.net). All sequencing data was released to Genbank with annotation and can be found using the BioProject ID as provided in Table 1.

## Availability and requirements

**Project name:** JCVI Primer Designer.

**Project home page:**http://sourceforge.net/projects/primerdesigner/.

**Operating system**: Tested and in production on Linux.

**Programming language:** Perl.

**License:** GNU GPL.

**Any restrictions to use by non-academics:** none.

## Competing interests

The authors declare that they have no competing interests.

## Authors’ contributions

TBS and KL conceived of the study and participated in its design. KL and AB developed algorithms and wrote the software. SS and VT designed appropriate consensus sequences, and computationally designed primers. JB, ATN and RH performed laboratory experiments, validated utility of results and generated data. SS and DK accumulated, organized, and generated PCR success rate statistics. KL and SS wrote the manuscript. All authors read and approved the submitted manuscript.

## Supplementary Material

Additional file 1 **This tab-separated values text file contains the amplicon name, forward and reverse primer pair sequences, estimated melting temperatures, sequencing success rates, and number of PCR attempts (isolates).** The amplicon name includes the virus name and start and stop positions on the template. PCR primer sequences were 5’-tailed with the forward and reverse M13 sequences, TGTAAAACGACGGCCAGT and CAGGAAACAGCTATGACC, respectively. Click here for file
